# The duplication mutation of Quebec platelet disorder dysregulates *PLAU*, but not *C10orf55*, selectively increasing production of normal *PLAU* transcripts by megakaryocytes but not granulocytes

**DOI:** 10.1371/journal.pone.0173991

**Published:** 2017-03-16

**Authors:** Catherine P. M. Hayward, Minggao Liang, Subia Tasneem, Asim Soomro, John S. Waye, Andrew D. Paterson, Georges E. Rivard, Michael D. Wilson

**Affiliations:** 1 Department of Pathology and Molecular Medicine, McMaster University, Hamilton, ON, Canada; 2 Department of Medicine, McMaster University, Hamilton, ON, Canada; 3 Hamilton Regional Laboratory Medicine Program, Hamilton, ON, Canada; 4 Genetics and Genome Biology, The Hospital for Sick Children, Toronto, ON, Canada; 5 Molecular Genetics, University of Toronto, Toronto, ON, Canada; 6 The Dalla Lana School of Public Health and Institute of Medical Sciences, University of Toronto, ON, Canada; 7 Hematology/ Oncology, Centre Hospitalier Universitaire Sainte-Justine, Montreal, QC, Canada; 8 Heart & Stroke Richard Lewar Centre of Excellence in Cardiovascular Research, Toronto, Canada; Ludwig-Maximilians-Universitat Munchen, GERMANY

## Abstract

Quebec Platelet disorder (QPD) is a unique bleeding disorder that markedly increases urokinase plasminogen activator (uPA) in megakaryocytes and platelets but not in plasma or urine. The cause is tandem duplication of a 78 kb region of chromosome 10 containing *PLAU* (the uPA gene) and *C10orf55*, a gene of unknown function. QPD increases uPA in platelets and megakaryocytes >100 fold, far more than expected for a gene duplication. To investigate the tissue-specific effect that *PLAU* duplication has on gene expression and transcript structure in QPD, we tested if QPD leads to: 1) overexpression of normal or unique *PLAU* transcripts; 2) increased uPA in leukocytes; 3) altered levels of *C10orf55* mRNA and/or protein in megakaryocytes and leukocytes; and 4) global changes in megakaryocyte gene expression. Primary cells and cultured megakaryocytes from donors were prepared for quantitative reverse polymerase chain reaction analyses, RNA-seq and protein expression analyses. Rapidly isolated blood leukocytes from QPD subjects showed only a 3.9 fold increase in *PLAU* transcript levels, in keeping with the normal to minimally increased uPA in affinity purified, QPD leukocytes. All subjects had more uPA in granulocytes than monocytes and minimal uPA in lymphocytes. QPD leukocytes expressed *PLAU* alleles in proportions consistent with an extra copy of *PLAU* on the disease chromosome, unlike QPD megakaryocytes. QPD *PLAU* transcripts were consistent with reference gene models, with a much higher proportion of reads originating from the disease chromosome in megakaryocytes than granulocytes. QPD and control megakaryocytes contained minimal reads for *C10orf55*, and C10orf55 protein was not increased in QPD megakaryocytes or platelets. Finally, our QPD megakaryocyte transcriptome analysis revealed a global down regulation of the interferon type 1 pathway. We suggest that the low endogenous levels of uPA in blood are actively regulated, and that the regulatory mechanisms are disrupted in QPD in a megakaryocyte-specific manner.

## Introduction

Urokinase-type plasminogen activator (uPA) is an important activator of fibrinolysis. The uPA from blood cells is important for fibrinolysis as bone marrow transplantation corrects the fibrinolytic defect of uPA deficient mice [1]. Normally, granulocytes contain most of the cellular uPA and fibrinolytic activity in blood [2, 3]. In contrast, platelets inhibit fibrinolysis by releasing large amounts of active plasminogen activator inhibitor 1 (PAI-1) but little uPA [4]. Inherited bleeding disorders that increase uPA in blood cells are rare: the only known example is Quebec platelet disorder (QPD), an autosomal dominant bleeding disorder with a unique, gain-of-function defect in fibrinolysis due to increased platelet uPA [5]. Persons with QPD suffer delayed-onset bleeding following hemostatic challenges unless treated with fibrinolytic inhibitors [6].

The fibrinolytic abnormalities of QPD are unique: the disorder selectively increases platelet uPA without triggering systemic fibrinolysis [4, 7, 8], due to overexpression of *PLAU* (the uPA gene) during megakaryocyte differentiation [5, 9, 10]. Although QPD increases the amount of uPA stored in platelets >100 fold [4], it has minimal effects on uPA in urine and plasma [4, 5, 11, 12]. Cultured megakaryocytes derived from peripheral blood CD34+ hematopoietic stem cells have been used to study the pathogenesis of QPD as these cells produce and store abnormally large amounts of uPA [9]. While QPD CD34+ progenitors contain normal to minimally increased levels of uPA mRNA and protein (consistent with a gene duplication), their differentiation and maturation into QPD megakaryocytes leads to >100 fold-increased levels of uPA mRNA and protein after 13–14 days of culture, which is very similar to the increases in uPA mRNA and protein that are detected in QPD platelets produced *in vivo* [4, 9]. In QPD megakaryocyte cultures, uPA levels increase significantly in the final stages of differentiation (i.e., by day 13–14 of culture), mirroring increases in von Willebrand factor (VWF), a protein that is normally produced in the later stages of megakaryocyte differentiation [9]. Studies of uPA localization in QPD megakaryocytes and platelets have shown extensive colocalization of uPA with alpha-granule proteins, unlike uPA labeling of control cells which is indistinguishable from background [9]. The alpha-granules of QPD subjects have a normal morphology, despite the proteolytic degradation of many alpha-granule proteins [13]. Immunoelectron microscopic analyses indicate that uPA is contained in QPD alpha-granules whereas it is undetectable in other structures and in control platelets [9]. In QPD megakaryocytes, uPA shows extensive colocalization (96% overlap) with the alpha-granule protein thrombospondin 1 in both the perinuclear region and cytoplasmic granular structures, consistent with its ongoing biosynthesis and processing for regulated release [9].

A better understanding of the genetic mechanisms underlying QPD would provide direct tissue-specific insights into how fibrinolysis is regulated and altered by disease. Persons with this disorder suffer from bleeding symptoms and delayed wound healing that respond well to treatment with fibrinolytic inhibitors [6], suggesting that alterations to uPA are central to QPD disease pathogenesis [5]. QPD is associated with proteolytic degradation of stored alpha-granule proteins due to intraplatelet activation of the fibrinolytic cascade, without evidence of systemic fibrinogenolysis [4, 7, 8, 12] and mice genetically engineered to overexpress uPA in megakaryocytes have a QPD-like bleeding disorder [14]. The increased expression of uPA by QPD megakaryocytes *in vivo* leads to markedly increased storage of uPA in alpha-granules, consumption of active PAI-1 in platelets and intra-platelet plasmin generation with secondary proteolysis of diverse alpha-granule proteins [4, 7]. The proteolysis of alpha-granule proteins in QPD is thought to occur late, following the uptake of plasminogen into megakaryocytes and/or platelets [9]. When activated, QPD platelets release large quantities of active two chain (tcuPA) and low molecular weight uPA (LMW uPA) [4] from their alpha-granules, which trigger further plasmin generation, leading to accelerated clot lysis [12].

QPD is linked to *PLAU* [10] and it is caused by a tandem duplication of a 78 kb region of chromosome 10 that contains two genes: *PLAU* and *C10orf55*, the latter being antisense to *PLAU* [15]. Persons with QPD show marked overexpression of *PLAU* from the disease chromosome in megakaryocytes, based on allele-specific, real-time, reverse transcription, polymerase chain reaction (qRT-PCR) analysis of expressed single nucleotide polymorphisms (SNP) [10], however, the effects of QPD on *C10orf55* have not been studied.

*C10orf55* functions are unknown, but could include *PLAU* regulation, based on large regions of complementary sequences. While uPA mRNA levels in QPD hematopoietic stem cells and saliva cells are consistent with *PLAU* duplication, the >100 fold increase in uPA mRNA and protein levels in day 13 cultured QPD megakaryocytes and in QPD platelets [4, 5, 7, 9, 10, 12, 15] greatly exceeds the expected increases caused by a tandem gene duplication [16]. We ask if this greater than expected increase in gene expression extends to *C10orf55*. While indirect evidence suggests that QPD platelets account for most of the increased uPA in QPD blood [12], QPD leukocytes have never been studied, and we evaluated them to provide additional insights into QPD molecular pathogenesis.

To further determine if QPD selectively dysregulates *PLAU* in megakaryocytes, we investigated if QPD leads to: 1) markedly increased levels of *PLAU* transcripts and uPA in leukocytes, and specifically, in cells of the granulocyte and monocyte lineages as like megakaryocytes, these cells are derived from myeloid differentiation of a common hematopoietic progenitor cell; 2) overproduction of specific *PLAU* transcripts by the disease chromosome; 3) altered levels of *C10orf55* mRNA and protein in megakaryocytes/platelets and/or leukocytes and 4) global gene expression differences in megakaryocytes. We found that QPD dysregulates *PLAU*, but not *C10orf55*, and selectively increases production of normal *PLAU* transcripts from the disease chromosome in megakaryocytes but not leukocytes. We also found that QPD is associated with downregulation of the interferon type 1 pathway in the megakaryocyte transcriptome.

## Materials and methods

The studies were conducted in accordance with the revised Helsinki Declaration with approval from the Hamilton Integrated Research Ethics Board and the Centre Hospitalier Universitaire Sainte Justine Research Ethics Board. All participants provided written informed consent.

### Subjects and sample collection

Peripheral blood samples (≤200 mL/donation) were collected by multiple donations from 7 QPD subjects and 10 controls (n = 1 unaffected QPD family member, n = 9 general population controls). Peripheral blood that was used to rapidly prepare total leukocyte RNA using LeukoLOCK™ Total RNA System kits (ThermoFisher Scientific, Mississauga, ON) was collected into 6 ml BD Vacutainer® tubes (BD Canada, Mississauga, ON) containing EDTA. For platelet and CD34+ cell isolation [9], peripheral blood was collected into sterile acid citrate dextrose (1:6 volume/volume) containing 3 μM aprotinin and 3 μM PGE1. For affinity isolation of peripheral blood leukocytes, blood was anticoagulated with 10 U/mL (final) unfractionated heparin (Sandoz Canada Inc., Boucherville, QC).

### Cell isolation

Blood platelets were isolated from the upper two thirds of platelet rich plasma (PRP) by double centrifugation (150 x *g* for 15 min, twice) to minimize leukocyte contamination. Platelets were pelleted (1200 x *g* for 12 min), washed once with ~30–40 mL of wash buffer (36 mM citric acid, 5 mM glucose, 5 mM KCl, 1 mM MgCl_2_, 103 mM NaCl, 0.2% BSA, 100 mU/mL apyrase, 1 μm PGE_1_, pH 6.5) before solubilizing samples for protein and molecular analyses.

For most experiments, peripheral blood leukocytes were harvested by immuno-magnetic separation using EasySep^TM^ Positive Selection Kits (STEMCELL^TM^ Technologies, Vancouver, BC, Canada) to isolate CD66b^+^ (granulocytes) and CD3^+^ (T-lymphocytes) cells from blood and CD14^+^ cells (monocytes) from buffy coats. Flow cytometry was used to validate that the method yielded acceptable purities (>90%) of harvested cells, as described [9]. Leukocytes were also rapidly isolated from whole blood onto leukodepletion filters using LeukoLOCK™ Total RNA System kits that rapidly stabilize leukocyte RNA.

Megakaryocytes were grown in liquid culture from peripheral blood CD34+ cells, as described [9], except StemSpan^TM^ SFEM II media and Megakaryocyte Expansion Supplement containing cytokines were used, as recommended by STEMCELL Technologies, and megakaryocytes were harvested on day 14. Harvested megakaryocytes were assessed for acceptable viability and differentiation by flow cytometry and ELISA, as previously described [9]. For each megakaryocyte harvest, uPA and VWF levels in culture media were assessed to verify that the cells had differentiated to produce and secrete large amounts of VWF and that QPD cultures contained marked increased uPA at harvest, unlike control samples [9].

### Preparation of samples for analysis

For protein analysis, cell pellets were solubilized in Tris-saline containing 1.0% Triton X-100 and protease inhibitors as described [9] (final: 10^9^ platelets/mL; WBC 10^6^/mL or 10^7^/mL). For RNA analysis, cell pellets of washed platelets, cultured megakaryocytes and affinity purified leukocytes were dissolved in TRIzol® Reagent (Life Technologies Inc., Burlington, ON, Canada) followed by total RNA isolation (as recommended by the manufacturer) then treatment with RNAse-free DNAse before confirming RNA quantity and quality [9, 10]. Leukocyte RNA was also prepared using LeukoLOCK™ Total RNA System kits as recommended by the supplier. DNA was isolated from EDTA anticoagulated blood as described [10]. Samples were stored at -80°C until analyzed.

### Protein analyses

ELISA kits were used to quantify uPA (Oncogene Biosciences kits that detect multiple forms of uPA, Cedarlane, Burlington, ON, Canada), C10orf55 (Cusabio Biotech assay, Cedarlane), VWF (Affinity Biologicals, Ancaster, ON, Canada) and TFPI2 (BosterBio, Pleasanton, CA). As platelets are far more abundant than leukocytes in blood, the percentage of platelet contamination in leukocyte preparations (collected at least twice/QPD donor) was estimated using each donor’s VWF data for platelets and affinity purified granulocytes, monocytes and T-lymphocytes. Affinity purified leukocytes were excluded from uPA determinations if their estimated platelet contamination (based on VWF levels) exceeded 15% and results were averaged if multiple samples from a donor were acceptable.

Cellular uPA was evaluated by sodium dodecyl sulfate polyacrylamide gel electrophoresis (8% polyacrylamide gels), followed by Western blotting, as described [4, 9] using the following detecting antibodies: primary: 1:2500 rabbit anti-human urokinase, affinity purified (Cedarlane; catalogue #PS074, RRID:AB_420269; http://antibodyregistry.org/search?q=ps074); secondary: 1:25,000 Peroxidase AffiniPure Donkey Anti–Rabbit IgG (Jackson ImmunoResearch Laboratories, West Grove, PA; catalogue #711-035-152, RRID:AB_10015282; http://antibodyregistry.org/search?q=711-035-152). Cellular uPA was analyzed non-reduced and reduced using the following quantities of lysate and purified proteins: platelet lysates (10^9^/mL): 5 and 15 μl; granulocyte lysates (10^7^/mL): 25 and 52 μl; monocyte lysates (10^7^/mL): 35 and 50 μl; purified proteins (scuPA from Sekisui Diagnostics, Lexington, MA; other sources of protein as published [4]): tcuPA: 7 and 86 ng; scuPA: 10 ng (reduced only); LMW uPA: 4 and 22 ng.

### Quantitative reverse transcription polymerase chain reaction

RT was performed using High-Capacity RNA-to-cDNA kits (Applied Biosystems, ThermoFisher Scientific, Waltham, MA) by incubating total RNA in a Thermocycler (37°C, 1 hour, 20 μl reaction volume) with recommended RT Kit components and 20 units RNasin® Plus RNase Inhibitor (Promega Corp., Madison, WI).

To evaluate *PLAU* expression, qPCR was performed using the Applied Biosystems ViiA™ 7 Real-Time PCR System and TaqMan® Gene Expression Assays (ThermoFisher Scientific; *PLAU*: Hs00170182_m1; *TFPI2*: Hs04334126_m1; rs4065: C__3155393_10; *TBP*: Hs0099999910_m1; *HPRT1*: Hs0099999909_m1; *GAPDH*: 4352934E) as recommended, using included primers. Simultaneous analysis of the endogenous control housekeeping genes glyceraldehyde 3-phosphate dehydrogenase (*GAPDH*), TATA box binding protein (*TBP*; for all cells except monocytes) and hypoxanthine guanine phosphoribosyl transferase 1 (*HPRT1*) was used to correct for potential variations in template RNA, RT and qPCR efficiencies. qPCR singleplex reaction mixtures included: 2 μL cDNA, 7.5 μL TaqMan® Gene Expression Master Mix (containing DNA polymerase), 0.75 μL 20× TaqMan® Gene Expression Assay reagents and 4.75 μL RNAse-free water. Assays were done in triplicate (duplicate if limited cDNA) in 96-well TaqMan® optical reaction plates, with no-template and no RT controls. Data were evaluated using ViiA™ 7 Software. *PLAU* expression, relative to the endogenous control genes *TBP* (leukocytes isolated with leukodepletion filters, granulocytes and megakaryocytes only), *GAPDH* and *HPRT1*, was calculated using the efficiency corrected method of Pfaffl [17, 18]. For rs4065 and *TFPI2*, samples were submitted to The Center for Applied Genomics (TCAG; The Hospital for Sick Children, Toronto, ON, Canada) for droplet digital PCR (ddPCR) [17], using *TBP* as an endogenous control for *TFPI2*.

#### RNA-seq

To evaluate transcripts generated by the region of the QPD duplication, including the QPD breakpoint, approximately 1 μg of total RNA/sample from 3 control and 3 QPD samples of day 14 cultured megakaryocytes and affinity purified granulocytes was prepared. The quality of total RNA samples was checked on an Agilent Bioanalyzer 2100 RNA Nano chip (Agilent Technologies, Santa Clara, CA) following recommendations. RNA library preparation was performed following the NEB NEBNext Ultra Directional Library Preparation protocol (New England Biolabs, Ipswich, MA). Briefly, 100–500 ng of total RNA was used as the input material and enriched for poly-A mRNA, fragmented into the 200-300-bases range for 4 minutes at 94°C and converted to double stranded cDNA, end-repaired and adenylated at the 3’ to create an overhang A to allow for ligation of Illumina adapters with an overhang T; library fragments were amplified under the following conditions: initial denaturation at 98°C for 10 seconds, followed by 10 cycles of 98°C for 10 seconds, 60°C for 30 seconds and 72°C for 30 seconds, and finally an extension step for 5 minutes at 72°C; at the amplification step, each sample were amplified with a different barcoded adapters to allow for multiplex sequencing. 1 μl of the final RNA libraries was loaded on a Bioanalyzer 2100 DNA High Sensitivity chip (Agilent Technologies) to check for size; RNA libraries were quantified by qPCR using the Kapa Library Quantification Illumina/ABI Prism Kit protocol (KAPA Biosystems, Wilmington, MA). Libraries were pooled in equimolar quantities and paired-end sequenced on a Rapid Run Mode flowcell with the V3 sequencing chemistry on an Illumina HiSeq 2500 platform (Illumina Inc., San Diego, CA) following Illumina’s recommended protocol to generate paired-end reads of 100-bases in length. Sequencing and library preparation were performed by TCAG. Two of the three QPD subjects provided both cell types.

RNA-seq libraries were sequenced to a depth of ~23 million paired-end 100 bp reads. Reads were dynamically trimmed based on quality using Trimmomatic [19] and aligned to hg19 (with RNA-STAR [20]) and to the QPD breakpoint [15]. GENCODE v19 [21] gene annotations were used as a reference transcriptome for alignment, as well as for the analysis of splice junction and exon usage with JunctionSeq [22]. Variant calling on RNA-seq data was performed according to the GATK version 3.2.2 best practices workflow for RNA-seq variants [23, 24]. Alignment to a custom genome containing the polymorphism rs2227574 (-/G) was used to correct for alignment biases due to the indel. RNA-seq data were visualized as depth normal signal using the UCSC genome browser. Raw fastq files were uploaded to the European Genome-phenome Archive (EGAS00001001840, center: McMaster), with controlled access as stipulated by subjects’ consent forms.

### Genotyping

To determine the rs2227574 allele associated with QPD, DNA from 38 QPD subjects (most collected for previous studies [10, 15]) was amplified using PCR (primers: F:TGCCACACAGAGTGGTCTTT; R:CTCTACCTCCCAAAGCTCCAT) and genotyped by Sanger sequencing, as described [10].

### Statistical analyses

The Mann Whitney U test was used to compare QPD and control protein and qPCR data. ANOVA were used to compare multiple cell types. p values <0.05 were considered significant. All tests were two sided.

Gene level raw counts for RNA-seq data were obtained using htseq-count with mode = intersection-strict and GENCODE V19 annotation [25]. Genes with average counts of <1 tag per library were not included in the analyses. Normalization and differential gene expression were done with DESeq2 [26] individually for each cell type. Only genes passing a transcriptome-wide Bonferroni corrected false discovery rate (FDR) of 0.05 (Wald test) were considered as differentially expressed. Analysis of exon and splice junction usage was performed between QPD and control megakaryocytes using JunctionSeq with default parameters for both read counting and differential usage analysis [22]. Gene set enrichment analysis was performed using GSEAPreranked, available through the GenePattern suite of tools [27] using default parameters. Genes were ranked by log2 fold-change values as determined by differential expression analysis.

## Results

### uPA protein and mRNA levels in QPD and control leukocytes

**Fig 1:**
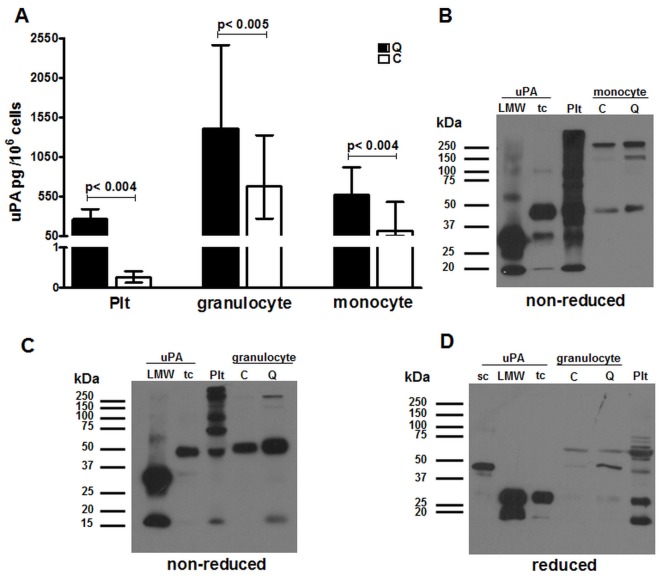
uPA in QPD and control leukocytes. A) Median uPA levels in granulocytes and monocytes compared to platelets (Plt) from QPD (n = 6) and control (n = 9) subjects (bars indicate ranges). B-D) Western blot images showing the forms of uPA visualized in QPD and control (respectively labeled Q and C) monocyte (B) and granulocyte (C, D) lysates, after separation by non-reduced (B, C) or reduced (D) SDS-PAGE, with comparison to two chain (tc), single chain (sc) and low molecular weight (LMW) uPA and uPA in QPD platelet lysates (Plt). S1 Supporting Information shows for reference the scans of original Western blots that were used to prepare panels B-D of Fig 1.

While indirect evidence suggests that QPD platelets account for most of the increased uPA in QPD blood [12], we studied QPD leukocytes to directly evaluate the cellular specificity of QPD molecular pathogenesis. In samples of affinity purified peripheral blood leukocytes with minimal platelet contamination (controls: undetectable; QPD ≤15%), QPD granulocytes and monocytes respectively contained about ~2 fold (p = 0.005) and ~5 fold (p = 0.004) more uPA than corresponding control cells, whereas QPD platelets from the same subjects contained >100 fold more uPA than control platelets (p = 0.004), as previously reported [4] (Fig 1). All subjects, including those with QPD, had more uPA in their granulocytes than monocytes, and the least uPA in their T-lymphocytes (amounts in T-lymphocytes: controls: <25 pg/10^6^ cells; QPD <25–65 pg/10^6^ cells, not shown).

Western blots indicated that the uPA in QPD and control leukocytes had the same mobility and confirmed that the amount of uPA was only modestly increased in QPD granulocyte and monocytes (Fig 1B and 1C). On non-reduced gels (Fig 1, panels B and C), one prominent form of uPA in all granulocyte and monocyte samples comigrated with recombinant tcuPA (which has an identical non-reduced mobility to scuPA) (4) and there were larger forms, likely complexed with protease inhibitor(s). On reduced gels, there were forms of uPA in QPD and control granulocytes that comigrated with tcuPA and with scuPA (Fig 1D). The amount of uPA in monocyte samples was insufficient for detection after reduced SDS-PAGE.

**Fig 2:**
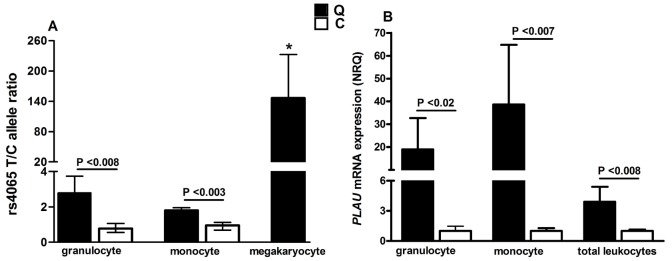
*PLAU* transcript levels in QPD and control leukocytes. Bars indicate standard errors and the p values compare data for QPD to control samples. A) T/C allele ratios for *PLAU* rs4065 were assessed for affinity purified granulocytes and monocytes from heterozygous QPD (n = 5) and control (n = 4) subjects, with simultaneous evaluation of QPD megakaryocytes as a known abnormal control (* indicates significantly higher values, p<0.0005, for QPD megakaryocytes compared to affinity isolated QPD leukocytes). B) *PLAU* mRNA expression levels (shown as NRQ) in affinity purified granulocytes and monocytes, and in total blood leukocytes that had been rapidly isolated using leukodepletion filters.

ddPCR analysis of the *PLAU* rs4065 (Fig 2A) indicated that the overexpression of *PLAU* by the disease chromosome was within the range expected for a gene duplication in both QPD granulocytes and monocytes (T/C allele ratios: ≤3.6), unlike the marked overexpression of *PLAU* by the disease chromosome in QPD megakaryocytes, which were included in the analyses as a known abnormal control (T/C ratio: >100, consistent with results previously reported [10])(p<0.05 by ANOVA). Because *ex vivo* manipulation of leukocytes is known to modify the transcript levels for many genes [28], we measured *PLAU* transcript levels in peripheral blood leukocytes that had been rapidly isolated with leukodepletion filters, and in granulocytes and monocytes that had been prepared by affinity purification which required more sample manipulations and multiple wash steps. Filter purified QPD peripheral blood leukocytes showed minimal increases in *PLAU* transcript levels compared to control samples (mean normalized relative quantities (NRQ): 3.9, Fig 2B) that were within the range expected for a gene duplication. *PLAU* transcript levels were higher in affinity purified QPD granulocytes and monocytes compared to control cells (respective NRQ: 19 and 39, Fig 2B), despite overlap in ΔCt values for individual samples (ranges, ΔCt values: granulocytes, QPD: -2.2 to 3.4, controls: -2.6 to 5.9; monocytes: QPD: 3.8 to 10.7, controls: 8.8 to 11.7)(Fig 2B). The greater increase in *PLAU* transcript levels in affinity isolated compared to filter purified leukocytes (Fig 2B), in the absence of increased uPA protein levels, suggested that leukocyte *PLAU* expression had been altered *ex vivo* during the longer affinity isolation procedure, and not by platelet contamination, as affinity purified QPD leukocytes did not show marked overexpression of *PLAU* by the QPD chromosome that was evident in QPD megakaryocytes (Fig 2A) and platelets [10].

### Megakaryocyte and granulocyte transcriptomes for the region that is duplicated in QPD

RNA-seq was used to evaluate the structure and allele-specific expression of *PLAU* and other transcripts derived from the duplicated region of chromosome 10, and flanking genes, in QPD and control megakaryocytes and to determine if marked overexpression of *PLAU* by the QPD disease chromosome altered transcript structure.

**Fig 3:**
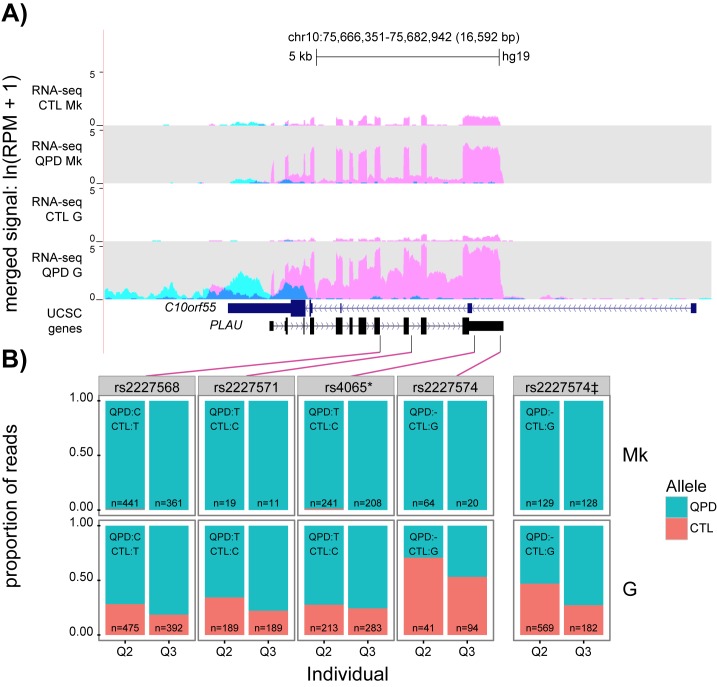
Comparison of gene expression at the *PLAU* locus in granulocytes and CD34+ derived megakaryocytes obtained using RNA-seq. A) UCSC genome browser screenshot showing normalized RNA-seq signal (tags per million) at the *PLAU* locus (merged for 3 individuals, for each cell and subject type; the signal tracks for individual samples are shown for reference in S1 Fig). Numbers indicate highest value (log scaled) within viewing range per track. Colors indicate reads that mapped to sense (pink) and antisense (blue) strands. Thick bars in the gene model represent exons, thin bars represent untranslated regions. *PLAU* variant 1 is shown. Chromosome position and genome assembly are displayed in the top track. B) Expression of SNPs in QPD megakaryocyte (top) and granulocytes (bottom). The proportion of reads mapping to control (red) and QPD (blue) alleles for cells from 2 QPD individuals with two alleles for each of the SNP shown, and n values indicate the total read coverage at each SNP. Only SNPs with > 10 mapped reads in each sample are shown. Purple lines delineate the position of individual SNP. * indicates SNP also evaluated in Fig 2. ^‡^ indicates allele ratios for rs2227574 after correction for alignment bias. CTL, control; Mk, megakaryocyte; G, granulocyte.

**Fig 4:**
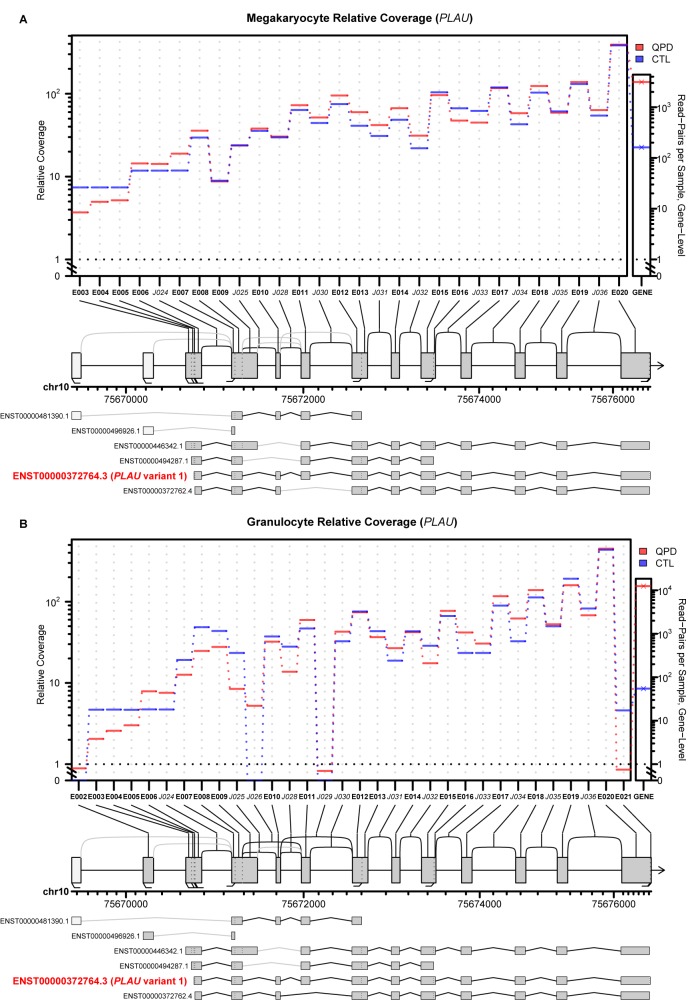
Exon and splice junction usage for *PLAU* in QPD versus control megakaryocytes and granulocytes, evaluated using JunctionSeq. The plots for megakaryocytes (A) and granulocytes (B) show the relative expression levels for each tested exon and splice junction, normalized to compensate for differences in gene expression, which are shown to the right as gene-level expression values. Vertical axis for the top panels are log-transformed except for the area between 0–1 which is plotted on a linear scale. The horizontal axis indicates the alpha numerical designations for exons (E) and junction sequences (J) for all annotated exons and splice variants in the Gencode v19 reference model. Only exons and junctions with coverage above the automatic detectable count threshold are plotted. The diagrams below indicate the positions of these elements on the full gene model that shows all annotated exons (boxes) and splice junctions (arcs). Exons and splice junctions which did not pass the automatic detectable count threshold are colored white and gray, respectively. The bottom panel compares the findings to all annotated *PLAU* isoforms, annotated as in the middle panel. The transcript model that corresponds to *PLAU* variant 1 transcript is indicated. Features showing significantly differential usage between QPD and control (CTL) samples, based on a criteria of p <0.05, are not colored-coded as none were detected.

In all subjects, most reads from the region of chromosome 10 that is duplicated in QPD came from *PLAU* transcripts (Fig 3 shows merged data by subject and cell type, for reference, S1 Fig shows the individual subject’s gene expression data that was used to prepare Fig 3). More than 20 fold increase in reads mapped to *PLAU* in QPD megakaryocytes than in control megakaryocytes (Fig 3). In contrast, the number of reads for the flanking genes, *CAMK2G* and *VCL*, were similar in QPD and control megakaryocytes (*CAMK2G*: 1.06 fold decrease, p>0.87; *VCL*: 1.16 fold increase, p>0.57; values for all genes reported in S2 Supporting Information), consistent with the report that QPD does not alter megakaryocyte *CAMK2G* or *VCL* expression [9]. No transcripts in QPD samples spanned the QPD breakpoint junction. No significant differences were detected in splice junction or exon usage for *PLAU* transcripts between QPD and control megakaryocytes (Fig 4). Of all annotated *PLAU* isoforms in GENCODE v19, only the isoform corresponding to *PLAU* transcript variant 1 (ENST00000372764.3) had reliable coverage across all annotated splice junctions in QPD and control megakaryocytes (Fig 4).

Allele-specific expression of *PLAU* was evaluated using RNA-seq reads that mapped to SNPs for the 2 QPD subjects for whom we obtained matched megakaryocyte and granulocyte samples. Consistent with ddPCR data (Fig 2), reads for rs4065 alleles in QPD granulocytes were detected in proportions consistent with an extra copy of the T allele on the disease chromosome (ratio: T:C ~ 3:1) whereas only the T allele was detected in the same subjects’ megakaryocytes (Fig 3). DNA genotyping indicated that all QPD subjects shared the rs2227574 deletion allele in the duplicated region and accordingly their genotypes were: —/- (n = 21) and—/G (n = 17). In samples from two QPD subjects that had the rs2227574 genotype—/G, reads for both alleles of this SNP were detected in their granulocytes (-/G ratio ~ 1.7:1, following correction for indel alignment bias) whereas only reads for the deletion allele were detected in their megakaryocytes (Fig 3). These QPD subjects showed similar biased expression in megakaryocyte but not granulocyte reads for rs2227568 (ratio: C:T ~ 3:1 in granulocytes and >100:1 in megakaryocytes) and for the *PLAU* intronic SNP rs2227571 (ratio T:C ~ 3:1 in granulocytes and >100:1 in megakaryocytes) (Fig 3).

**Fig 5:**
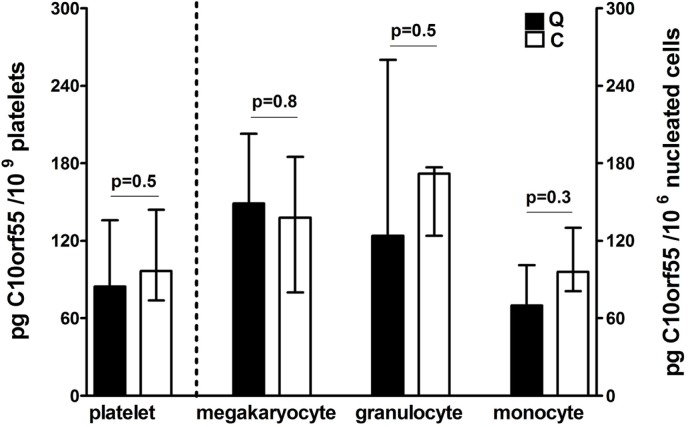
C10orf55 protein levels in QPD and control cells. ELISA data (medians, bars indicate ranges) comparing data for platelet, cultured megakaryocyte, granulocyte and monocyte lysates from QPD (Q, n = 6) and control (C; n = 6) subjects.

Reads originating from the DNA strand antisense to *PLAU* represented only ~1–9% of all reads at the *PLAU* locus in all samples evaluated by RNA-seq (Fig 3 shows merged data and individual sample data is shown for reference in S1 Fig) and there was very little C10orf55 protein in platelets, megakaryocytes and granulocytes from QPD and control subjects (Fig 5; p values ≥0.34 for QPD vs. control sample comparisons). In megakaryocytes, most antisense reads (~85%) mapped to the 3’UTR of *C10orf55* whereas in granulocytes, a substantial fraction of antisense reads (~10–45%) mapped to unannotated regions downstream of *C10orf55* (Fig 3, S1 Fig and S2 Fig). Very few reads mapped to other annotated exons of *C10orf55* in either cell type (Fig 3, S1 Fig and S2 Fig). Furthermore, none of the reads antisense to *PLAU* actually spanned annotated splice junctions, including the junction between the annotated transcription start site (penultimate exon) and the final exon (S2 Fig). The longest *C10orf55* open reading frame predicted from the final exon alone was in-frame and predicted to generate a 127 amino acid protein (S2 Fig, panel C).

#### Global expression differences in QPD megakaryocytes

**Fig 6:**
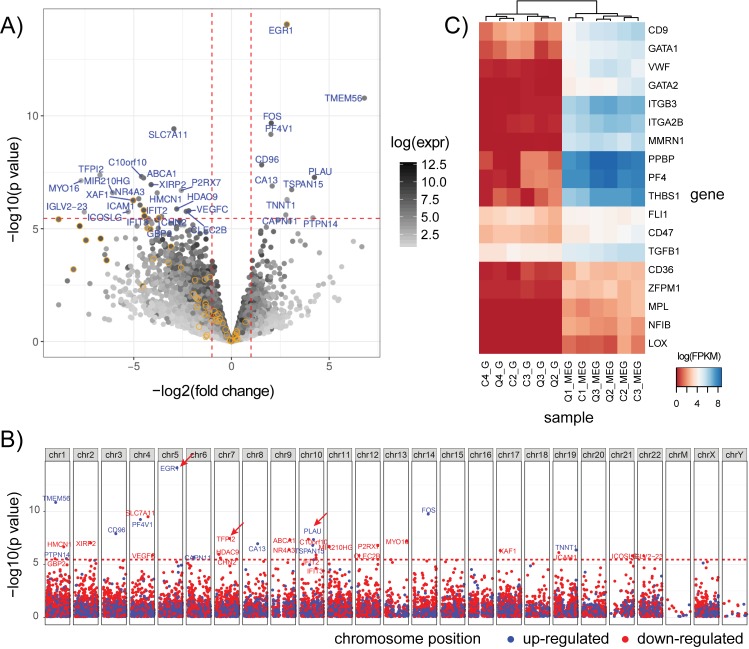
Global expression differences in QPD megakaryocytes. A) Differential expression (DE) analysis of genes, comparing QPD to control megakaryocytes. Log transformed p-values and fold-changes are shown on the vertical and horizontal axes, respectively. Horizontal dashed lines delineate the Bonferroni corrected FDR threshold of 0.05. Vertical dashed lines delineate a fold change of +/- 2 fold. Genes are shaded based on their mean expression level. Only genes exceeding the FDR and fold change cutoffs are labeled. Genes annotated as involved in type-1 interferon signaling (GO:0034340) are outlined in yellow. B) Manhattan plot of DE analysis for megakaryocytes. Genes are plotted based based on chromosomal position and significance. Genes in red are down-regulated in QPD and those in blue are up-regulated in QPD. Dashed red lines delineate the Bonferroni corrected FDR threshold of 0.05. Arrows mark the positions of *PLAU*, *EGR1 and TFPI2*. C) Unsupervised clustering of log transformed FPKM (fragments per kilobase of transcript per million mapped reads) values for a panel of literature-reported megakaryocyte marker genes for both megakaryocyte and granulocyte samples from individual QPD (Q) and control (C) subjects. Genes and samples were clustered based on Euclidean distance using the hclust function in R with default parameters. Markers were selected as described in [31].

Although our study was designed to look at *PLAU* splice junction usage and C10orf55 levels in QPD, we evaluated if there were significant global differences in megakaryocyte gene expression in QPD. Differential expression (DE) analysis of control versus QPD megakaryocytes identified a total of 32 significantly DE genes (Bonferroni corrected p-values < 0.05) (Fig 6A) that did not show clustering by physical location (Fig 6B). Among the DE genes (Fig 6A), the genes with implications for hemostasis included *PLAU* and *TFPI2*, a protease inhibitor with activity against plasmin [29, 30] that showed ~ 100 fold decreased expression in QPD megakaryocytes (Fig 6A, signal tracks shown for reference in S3 Fig). On the other hand, some other DE genes, such as *EGR1*, showed increased expression in QPD megakaryocytes (Fig 6A, signal tracks shown for reference in S3 Fig). Some DE genes were of uncertain biological significance given their low levels of expression in normal megakaryocytes and/or unknown functions (e.g., *TMEM56*)(Fig 6A). We further examined the expression of a number of literature-reported megakaryocyte marker genes [31] and megakaryocyte-synthesized alpha-granule proteins [32] as a way to assess our megakaryocyte cultures (Fig 6C). These included: megakaryocyte-associated proteins (e.g., *LOX*), megakaryocyte-erythroid transcription factors (e.g., *GATA1*, *GATA2*, *FLI1*, *NFIB*), cell surface markers (e.g., CD41/*ITGA2B*, CD9, CD61/*ITGB3*), and alpha-granule proteins (e.g., transforming growth factor beta 1/*TGFB1*, *VWF*, thrombospondin-1/*THBS1*, platelet factor IV/*PF4*, beta-thromboglobulin/*PPBP*, multimerin 1/*MMRN1*). Megakaryocyte markers showed elevated expression in megakaryocytes versus granulocytes and did not significantly differ between QPD and control samples (Fig 6C). Furthermore, unsupervised clustering of samples based on marker gene expression did not result in the separation of QPD and control samples, suggesting that the *in vitro* differentiation was comparable between control and QPD cultured megakaryocytes (Fig 6C). Gene set enrichment analysis of DE genes in control vs QPD megakaryocytes identified an enrichment for genes involved in type I interferon response and the immune response, the majority of which had reduced expression in QPD versus control megakaryocytes (S2 Supporting Information). In contrast, no enrichment for interferon response genes was observed for control versus QPD granulocytes.

The only interferon response gene that showed increased expression in QPD megakaryocytes was the early growth response gene *EGR1*, which was ~ 8-fold upregulated compared to controls (Fig 6A). *EGR1* was highly expressed in QPD and control granulocytes, but it did not show DE in these cells (1.84 fold increase, p>0.29; S3 Fig). Upregulation of *EGR1* in QPD megakaryocytes is interesting as *EGR1* has been implicated in the regulation of *PLAU* in osteosarcomas [33]. Thus, we used our RNA-seq data to ask whether *EGR1* transcripts differed in terms of splicing or coding sequence in QPD as these could potentially influence PLAU expression. However, we did not find any coding variants nor significant differences in splice junction usage between QPD and controls for *EGR1* (S3 Fig).

**Fig 7:**
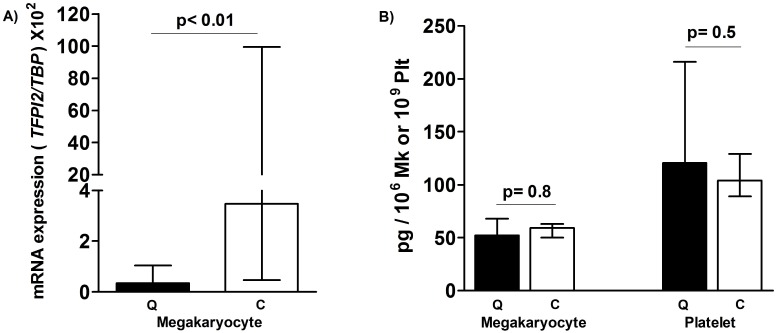
*TFPI2* mRNA and protein levels in QPD and control megakaryocytes and platelets. A) *TFPI2* ddPCR findings for cultured megakaryocytes from QPD (Q, n = 6) and control (C; n = 6) subjects. B) TFPI2 ELISA findings (medians, bars indicate ranges) for platelets and cultured megakaryocytes from QPD (Q, n = 6) and control (C; n = 6) subjects. p values shown compare QPD to control findings.

Given that *TFPI2* is a plasmin inhibitor [29, 30], and its downregulation might make the QPD fibrinolytic defect worse, we took additional steps to validate differences in *TFPI2* mRNA and protein levels using ddPCR and ELISA. While we did detect and validate that there is a significant decrease in *TFPI2* mRNA in QPD versus control megakaryocytes using ddPCR (Fig 7A), there were no significant reductions in TFPI2 protein in either QPD megakaryocytes or platelets, relative to control samples (Fig 7B). RNA-seq read coverage for *TFPI2* in QPD megakaryocytes (S3 Fig) was insufficient for the analysis of splice junctions and coding variants.

## Discussion

The molecular mechanism underlying the much greater than expected increases in both uPA mRNA and protein during QPD megakaryopoiesis is not well understood (12). As other hematopoietic lineages, and the other duplicated gene, *C10orf55*, had not been studied in QPD, we further explored the nature of the *PLAU* regulatory defect in this bleeding disorder. In our analysis of *PLAU* and *C10orf55* transcripts in leukocytes and megakaryocytes, we found direct evidence that QPD leads to a selective, megakaryocyte-specific, overexpression of *PLAU* by the disease chromosome. QPD did not increase uPA in leukocytes of myeloid and lymphoid lineages beyond the levels expected for a gene duplication, nor did it alter C10orf55 levels in megakaryocytes or leukocytes. First, both RT-qPCR and RNA-seq analyses indicated that QPD leukocytes expressed both *PLAU* alleles, in proportions consistent with *PLAU* duplication on the disease chromosome, in contrast to QPD megakaryocytes which almost exclusively expressed *PLAU* from the disease chromosome. Second, there were minimal increases in *PLAU* mRNA in leukocytes rapidly isolated with kits to stabilize total leukocyte RNA (NRQ, QPD vs. controls: ~3.9). Third, affinity purified QPD granulocytes and monocytes contained minimally increased uPA (levels: ~2–5 fold greater than controls), unlike QPD megakaryocytes and platelets (levels: >100 fold greater than controls). Fourth, we observed an abundance of *PLAU* intronic reads in affinity purified QPD granulocytes, which is known to occur during acute gene transcription [34, 35] (Fig 3, S1 Fig).

By observing a ~3:1 ratio of expression of the disease compared to non-disease alleles of *PLAU* SNPs in the granulocytes of QPD individuals with two alleles, we conclude that *PLAU* expression in perturbed QPD granulocytes occurs through an independent regulatory mechanism. Taken together, our findings illustrate that megakaryocytes/platelets are the only QPD blood lineage that, *in vivo*, overexpresses *PLAU* beyond the levels expected for a gene duplication. While QPD also duplicates *C10orf55*, there was little C10orf55 protein in the megakaryocytes and platelets, or leukocytes, from all subjects and few reads for *C10orf55* were detected by RNA-seq. Importantly, these data provide new direct evidence that a megakaryocyte and *PLAU* specific defect in gene regulation underlies the marked over-expression of *PLAU* by the disease chromosome in QPD, which is the hallmark feature of this unique bleeding disorder.

RNA-seq analyses did not detect evidence of differential splice junction usage for *PLAU* between QPD and control megakaryocytes and granulocytes. The patterns of detectable splice junction and exon usage suggest that the most abundant *PLAU* isoform in all megakaryocyte and granulocyte samples corresponds to the *PLAU* variant 1 transcript that encodes scuPA. Additionally, no reads in QPD megakaryocytes or granulocytes crossed the QPD breakpoint junction. These data concur with our earlier observations that QPD platelet uPA is functional and has a normal mobility (consistent with its activation to tcuPA, LMW uPA and ability to form complexes with PAI-1) (4) and our new observations that the uPA in QPD granulocytes and monocytes also had a normal mobility. We did observe some differences in the non-reduced and reduced profile of QPD platelet and leukocyte uPA, likely due to cell-type specific differences in extent of uPA proteolysis and in sizes of proteins that formed complexes with uPA (Fig 1). The observation that *PLAU* over-expression by the QPD disease chromosome increased levels of normal *PLAU* transcripts has important implications for the megakaryocyte specific regulatory mechanism that increases uPA >100 fold in QPD megakaryocytes and platelets.

Previous comparisons of uPA levels in QPD platelets versus plasma, and in serum harvested after an overnight incubation, suggested that QPD platelets are the major source of uPA in QPD blood [4, 12]. Based on the average cellular uPA levels that we measured in leukocytes, and subject blood counts, we estimate that granulocytes and monocytes normally contain ~99% of the blood cellular uPA, whereas in QPD, platelets contain the majority (~86%). The measured quantities of uPA in leukocytes could be an underestimate, if the affinity purification of peripheral blood leukocytes triggered some intracellular uPA release. Our data provide quantitative insights on earlier observations that in normal blood, most uPA activity is contained within polymorphonuclear cells (i.e., granulocytes) [2, 36]. We confirmed earlier reports that granulocyte and monocyte uPA have the same mobility as scuPA [36–38], although we also detected additional, high molecular weight forms of uPA in these cells, and a form of uPA with the mobility of tcuPA in QPD and control granulocytes.

There is little known about *C10orf55*, which encodes an uncharacterized protein, C10orf55 [39, 40]. As 2928 bp of *C10orf55* are antisense to spliced *PLAU* transcripts, it is possible that *C10orf55* transcription, or the transcript itself, could potentially influence *PLAU* expression. However, since we did not detect significant differences in the reads for *C10orf55* RNA in QPD and control megakaryocytes, and most mechanisms described to date for cis-natural antisense transcripts involve inhibition (i.e., through transcriptional interference) [41], the duplication of *C10orf55* does not explain the increased *PLAU* expression in QPD. We detected no reads spanning annotated splice junctions in the *C10orf55* gene model (GENCODE v19) [21]. As the vast majority of observed reads antisense to *PLAU* in QPD granulocytes did not map to the annotated exon of *C10orf55* (thick bar in gene model, Fig 3), but rather, to the 3’UTR region, the antisense reads could be non-coding RNA produced as a result of bidirectional transcription at the *PLAU* promoter. It is possible that the very low levels of C10orf55 protein that we detected in both QPD and control megakaryocytes, platelets and leukocytes could represent synthesis of a truncated protein or alternatively, non-specific interferences from other proteins on the measurement of C10orf55 by the commercial ELISA.

Our observation that there were minimal increases in *PLAU* transcript levels in QPD compared to control total blood leukocyte RNA (prepared after rapid filter capture), that were within the range expected for a gene duplication [16], illustrate that QPD causes lineage-specific, marked overexpression of *PLAU* expression by megakaryocytes but not by leukocytes. The handling of blood samples *ex vivo* is known to alter leukocyte transcripts, and induce expression of some genes [28]. It was interesting that the lengthier, affinity purification of granulocytes (which are the most abundant type of leukocyte in blood) increased *PLAU* expression by both QPD chromosomes *ex vivo*, implicating a different mechanism than the defect that upregulates *PLAU* expression in QPD megakaryocytes *in vivo*. The greater platelet contamination of QPD leukocyte preparations, despite the lower QPD platelet counts, likely reflects proportionately more platelet attachment to leukocytes in QPD samples despite their processing with inhibitors of fibrinolysis and platelet activation. As we excluded leukocytes samples with ≥15% platelet contamination when analyzing uPA, and as platelets are much smaller and have a much lower RNA content than leukocytes [42], we suggest that there was little interference from QPD platelets in the leukocyte uPA mRNA and protein estimates. Furthermore, our affinity purified granulocytes showed very low expression of megakaryocyte/platelet markers, such as *ITGA2B* and *ITGB3*. It is nonetheless possible that increased plasmin generation during the processing of QPD samples increased platelet activation, platelet adhesion to leukocytes, and leukocyte activation, as plasmin is known to activate platelet and leukocytes, and activated platelets bind to leukocytes [43–45].

The analysis of transcriptome-wide changes with RNA-seq identified modest differences in gene expression between control and QPD megakaryocytes. Notably, canonical megakaryocyte markers and transcripts for megakaryocyte-produced alpha-granule proteins did not differ significantly. These observations are consistent with the previous finding that alpha-granule protein changes in QPD are due to proteolytic degradation rather than altered expression. The observed differences in the expression of *TFPI2* in megakaryocytes is of potential interest as *TFPI2* is a plasmin inhibitor [30]. However, the lack of detectable changes in TFPI2 at the protein level in QPD platelets or megakaryocytes suggests that altered *TFPI2* expression is unlikely to have major contributions to QPD pathology. While it is possible that *TFPI2* expression in other cell types may be of relevance, RNA-seq data from 8 blood cell types recently profiled by the BLUEPRINT consortium [46] suggests that *TFPI2* expression in blood cells is limited to megakaryocytes and hematopoietic progenitor cells. Likewise, we did not detect reads for *TFPI2* in any of our granulocyte RNA-seq datasets. Gene set enrichment analysis revealed an enrichment of genes involved in type I interferon signaling, amongst genes showing decreased expression in QPD versus control megakaryocytes. Given that both urokinase-plasminogen receptor (uPAR) and type I interferon signaling act through JAK-activation of STAT1/STAT2 to initiate transcriptional responses [47–49], we speculate that overexpression of *PLAU* and consequent uPAR activation may lead to feedback control of STAT responsive gene expression. The increased expression of the transcriptional regulator *EGR1* in QPD megakaryocytes is interesting as *EGR1* is involved in the regulation of uPA, and overexpression of *EGR1* has been shown to down-regulate uPA and uPAR in osteosarcomas [33]. It is possible that elevated expression of *EGR1* in QPD reflects a feedback mechanism to compensate for increased levels of uPA and uPAR signaling. Interestingly, we did not observe significant differences in the expression of *EGR1* nor other interferon response genes between QPD and control granulocytes. One possible explanation is that these changes in the expression of *TFPI2*, *EGR1* and other genes in QPD megakaryocytes are due to long-term exposure to high (>100-fold increased) levels of uPA in QPD megakaryocytes [9]. Our analyses showed that transcript structure of *EGR1* is normal in QPD megakaryocytes and unlikely to explain differences in transcript abundance. We had insufficient reads for similar analysis of *TFPI2* in QPD megakaryocytes. However, as individuals with QPD are closely related, we cannot rule out that genetic factors other than the *PLAU* duplication contributed to the observed differences in the expression of interferon response genes and other genes (e.g., *TFPI2*) between QPD and control subjects. The modest differences between the QPD and control subject populations support that: a) our cultured megakaryocyte differentiation procedure was robust; b) we had the power to detect changes in lowly expressed megakaryocyte genes; and c) that the phenotype of QPD megakaryocytes is similar to non-affected individuals at the level of the transcriptome. While more replicates and megakaryocytes from unaffected family members would be necessary to further study the biological effect of increased *PLAU* on megakaryocytes, our results underscore the clinical observation that QPD megakaryocytes are phenotypically normal apart from their fibrinolytic defect.

Our current study provides important new direct evidence that the QPD gene regulatory defect specifically and selectively dysregulates *PLAU*, but not *C10orf55*, by a mechanism that is specific to megakaryocyte, but not granulocyte or monocyte differentiation, and selectively increases production of normal *PLAU* transcripts (but not *C10orf55* transcripts) from the disease chromosome beyond the level expected for a gene duplication mutation. Further clues about the molecular pathogenesis underlying QPD will likely require a detailed epigenomic analysis of the *PLAU* locus in QPD and control megakaryocytes. Future studies will need to compare epigenomic findings for QPD megakaryocytes to QPD hematopoietic cells that do not show marked overexpression of *PLAU* by the disease chromosome, to help identify the regulatory changes that are unique to QPD megakaryocytes.

## Conclusions

The QPD duplication mutation selectively dysregulates *PLAU* in megakaryocytes but not in leukocytes, leading to increased production of normal *PLAU* transcripts by megakaryocytes that far exceed the modest increases expected for a gene duplication mutation. The observation that QPD dysregulates *PLAU* but not *C10orf55* (the other duplicated gene) in megakaryocytes, and that QPD increases production of normal *PLAU* transcripts from the disease chromosome in megakaryocytes [1], provides further evidence that a megakaryocyte specific defect that selectively upregulates *PLAU* transcript levels underlies the pathogenesis of QPD.

## Supporting information

S1 Fig Genome browser view of RNA-seq data for the *PLAU* locus, showing the individual sample tracks for the data presented in Fig 3A. Top: individual signal tracks (normalized as reads per million) are shown at a fixed (log transformed) scale. Bottom: Same as top panel, except scales are adjusted to reflect the highest value within the viewing range. Paired samples from the same individual have the same numeric identifier.
(EPS)

S2 Fig Exon and splice junction usage for *C10orf55* in megakaryocyte and granulocyte samples. Panels A and B compares data for QPD vs control megakaryocytes (A) and granulocytes (B) evaluated using JunctionSeq, with the relative expression level for each tested exon and splice junction normalized to compensate for differences in gene expression, which are shown as gene-level expression values. Data were plotted as described in the legend to Fig 4. Only exons and splice junctions with coverage above the automatic detectable count threshold are plotted. No features were observed to be significantly different between QPD and control for either cell type. Numeric values for mean coverage, dispersion, and p-values are reported for all annotated exons and splice junctions in Tables C and D of S1 Supporting Information. Panel C shows the predicted protein sequence of C10orf55, based on Genbank accession AIC53846.1. // denotes junction point between final exon.
(EPS)

S3 Fig Expression of *TFPI2* and *EGR1* and splice junction analysis of *EGR1* in megakaryocyte and granulocyte samples. Panels A and B show UCSC genome browser sessions of normalized RNA-seq signal for the *TFPI2* and *EGR1* genes. Normalization, samples, and color coding as described in the legend to Fig 3A. Signal for *TFPI2* is shown on a linear scale. Panel C shows junctionSeq analysis of *EGR1* for megakaryocytes and granulocytes (reads were insufficient for similar analysis of *TFPI2*). Axes and labels are as described in the legend to Fig 4.
(EPS)

S1 Supporting Information This supplement contains the original scanned images of the Western blots that were used to prepare the panels B-D of Fig 1. (PDF)

S2 Supporting Information DEseq2 and GSEA results for DE analysis of QPD versus control megakaryocytes. This spreadsheet contains 6 tables. Tables A and B contain the results of DE analysis with DESeq2 for QPD versus control megakaryocytes and granulocytes, respectively. Tables C-F contain the gene set enrichment analysis results using for top 10 enriched pathways/terms for downregulated and upregulated genes in megakaryocytes and granulocytes, respectively.
(XLSX)
