# Supplementary material for: The duplication mutation of Quebec platelet disorder dysregulates PLAU, but not C10orf55, selectively increasing production of normal PLAU transcripts by megakaryocytes but not granulocytes
Source: PLoS One. 2017 Mar 16;12(3):e0173991. doi: 10.1371/journal.pone.0173991 (PMC5354430; doi:10.1371/journal.pone.0173991)

S1 Supporting Information.

This supplement contains the original scanned images of the Western blots that were used to prepare the panels B-D of Fig 1.

Panel B scan below shows a non-reduced gel, analyzed by Western blotting for uPA. The lanes (right to left) show uPA in: monocytes (M)from QPD (Q) and control (C) subjects, QPD platelets (Plt), and in preparations of two chain (tc) and low molecular weight (LMW) uPA that were run as additional controls. The positions of prelabeled markers are also shown. The black dot at the lower right was used to mark the orientation.

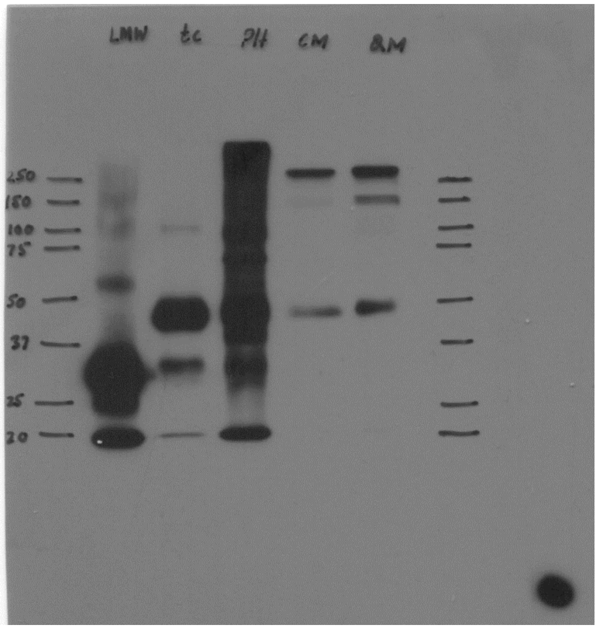

Panel C scan below shows a non-reduced gel, analyzed by Western blotting for uPA. The lanes (right to left) show uPA in: granulocytes (G) from QPD (Q) and control (C) subjects, QPD platelets (plt), and in preparations of two chain (tc) and low molecular weight (LMW) uPA that were run as additional controls. The positions of prelabeled markers are also shown. The two black dots at the lower right were used to mark the orientation.

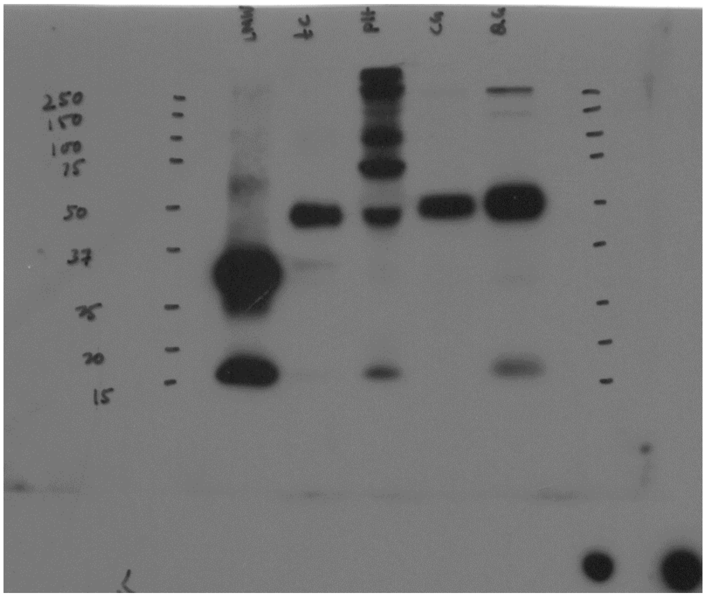

Panel D scan below shows a reduced gel, analyzed by Western blotting for uPA. The lanes (right to left) show uPA in: granulocytes (G) from QPD (Q) and control (C) subjects, QPD platelets (plt), and in preparations of two chain (tc), low molecular weight (LMW) and single chain (sc) uPA that were run as additional controls. The positions of prelabeled markers are also shown. The black dots at the upper left and at the lower right were used to mark the orientation.

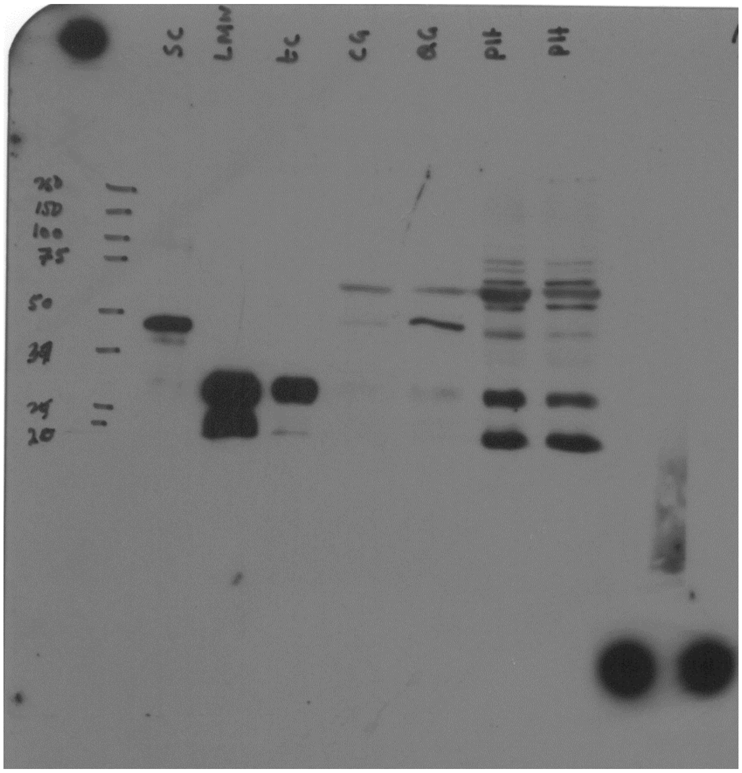

Supplement: S1 Supporting Information — (PDF) [file pone.0173991.s004.pdf]
